# Longitudinal Assessment of Joint Health in Paediatric Haemophilia Using the HEAD-US Ultrasound Scoring System: The ULTRA Study

**DOI:** 10.3390/life16061000

**Published:** 2026-06-14

**Authors:** Aikaterini Michalopoulou, Olympia Papakonstantinou, Athina Dettoraki, Dimitrios Syrengelas, Vassiliki Bizimi, Konstantina Dakou, Miltiades Kyprianou, Vassiliki Spoulou, Helen Pergantou, Christina Kanaka-Gantenbein

**Affiliations:** 1 Haemophilia Centre/Haemostasis and Thrombosis Unit, “Aghia Sophia” Children’s Hospital, GR11527 Athens, Greece; katia.michalopoulou@gmail.com (A.M.); athinadett@gmail.com (A.D.); kos.dakou@gmail.com (K.D.); miltosk@gmail.com (M.K.); 2 2nd Department of Radiology, “Attikon” University Hospital, National and Kapodistrian University of Athens, GR11527 Athens, Greece; sogofianol@gmail.com (O.P.); bizimi@otenet.gr (V.B.); 3 Department of Pediatric Physical Therapy, “Aghia Sophia” Children’s Hospital, GR11527 Athens, Greece; syrengelasd@gmail.com; 4 First Department of Pediatrics, School of Medicine, National and Kapodistrian University of Athens, GR11527 Athens, Greece; vspoulou@med.uoa.gr (V.S.); ckanaka@med.uoa.gr (C.K.-G.)

**Keywords:** haemophilia, arthropathy, ultrasound, HEAD-US, paediatric, synovitis

## Abstract

**Background:** Early detection of joint damage in haemophilia is essential to prevent haemophilic arthropathy. This study longitudinally evaluated joint health in paediatric patients with haemophilia in Greece using the Haemophilia Early Arthropathy Detection with Ultrasound (HEAD-US) scoring system. **Methods:** Children aged 5–18 years with haemophilia of any severity, all receiving prophylaxis, were assessed at baseline and during longitudinal follow-up visits scheduled at 6, 12, 24, and 36 months. Evaluations included HEAD-US and Haemophilia Joint Health Score (HJHS), functional ability (PedHAL), health-related quality of life (HRQoL), and pain (BPI). **Results:** Forty-seven boys (mean age 11.5 ± 3.7 years) were included (severe 83%, moderate 11%, mild 6%). Baseline HEAD-US scores were low (mean ± SE: 0.22 ± 0.069). No significant overall change was observed over time, although left knee showed significant improvement (*p* < 0.05). Ankles consistently exhibited higher scores compared to other joints (*p* < 0.05). Synovitis was the most frequent abnormality, while cartilage damage was infrequent and subchondral bone changes were negligible. HEAD-US detected more abnormalities than HJHS. **Conclusions:** Joint health was well preserved under prophylaxis, with synovitis representing the earliest detectable change, although interpretation is limited by incomplete long-term follow-up. HEAD-US proved more sensitive than clinical assessment, supporting its role in routine longitudinal monitoring.

## 1. Introduction

Haemophilia A and B are rare, inherited X-linked bleeding disorders resulting from partial or complete deficiencies of coagulation factors VIII (FVIII) or IX (FIX)caused by *F8* (haemophilia A) or *F9* (haemophilia B) gene mutations on the X chromosome [1]. Both haemophilia A and haemophilia B are recessive disorders affecting almost exclusively males. Females are typically heterozygous carriers of a single mutated gene and may exhibit reduced FVIII or FIX levels, which are usually associated with mild clinical symptoms [2]. Disease-severity classification is based on the amount of residual FVIII or FIX activity: severe (<1 international unit (IU)/dL), moderate (1–5 IU/dL) or mild (>5–40 IU/dL) [3]. Male haemophilia A prevalence has been traditionally estimated at one in 5000 live births, and that of haemophilia B at one in 30,000 live births, whereas a 2019-report suggested higher prevalence (one in 4000 male live births for haemophilia A and one in 20,000 male live births for haemophilia B) [4,5].

Spontaneous joint bleeding (haemarthrosis) is the most frequent clinical manifestation in adults and children with severe haemophilia; however, it may also occur in patients with moderate or mild disease [6]. Most bleeding episodes in haemophilia patients occur in mechanical or weight bearing joints (knees, elbows and ankles),which may ultimately lead to painful and disabling haemophilic arthropathy and reduced health-related quality of life (HRQoL) [2,6]. Prophylaxis with FVIII or FIX replacement therapy, or non-factor agents, is currently the standard-of-care treatment that has offered patients with haemophilia a near-normal lifespan with reduced levels of joint damage and morbidity [1,3,7]. On-demand (episodic) treatment is another therapeutic approach described as the use of replacement therapy in response to a bleed or when there is an imminent risk of bleeding; nevertheless, it is inferior to prophylactic treatment in preventing joint disease [1,6,8].

Joint health can be evaluated through a range of examinations, including clinical/physical assessments such as the Haemophilia Joint Health Score (HJHS), and imaging-based techniques, notably ultrasound scoring systems like the Haemophilia Early Arthropathy Detection with Ultrasound (HEAD-US) score [9,10,11]. The HEAD-US method enables direct evaluation of joint condition, allowing the identification of soft tissue damage and peripheral cartilage changes associated with early arthropathy, when interpreted alongside medical history and physical examination. It was designed as a longitudinal monitoring tool for the assessment of joint status, treatment effectiveness, and clinical outcomes and is intended for use as a point-of-care method by healthcare professionals without excessive imaging expertise [9]. Both physical examination and musculoskeletal ultrasound imaging have proven to be valuable tools for assessing joint health in individuals with haemophilia, as they provide distinct yet complementary information regarding haemophilic arthropathy [12].

Although several cross-sectional studies have demonstrated the utility of HEAD-US in detecting early haemophilic arthropathy, longitudinal real-world paediatric data combining ultrasound, clinical joint assessment, and patient-reported outcomes remain limited, particularly in children receiving contemporary prophylactic regimens [13,14,15,16,17,18,19].

The primary aim of this study was to longitudinally record the joint health of paediatric patients with haemophilia of different severity degrees, in Greece, using a systematic evaluation with point-of-care ultrasound. Secondary objectives included evaluation of arthropathy through HJHS measurements, functional ability and patient QoL assessments using patient reported outcomes (PROs) tools.

## 2. Methods

### 2.1. Study Design and Participants

This single-centre, prospective non-interventional study was conducted at the largest comprehensive paediatric haemophilia centre in Greece, the Haemophilia Centre/Haemostasis and Thrombosis Unit of ‘Aghia Sophia’ Children’s Hospital, Athens. Data were collected between March 2021 and September 2024.

Eligible patients were between 5 and 18 years of age, diagnosed with haemophilia A or B of any severity. Patients were excluded if they had surgery in any of the following joints (left/right ankle, left/right knee, left/right elbows) in the year prior to inclusion, were participating in an interventional study in the three months prior to inclusion, and/or were taking an Investigational Medicinal Product in the three months prior to inclusion.

### 2.2. Procedures and Study Assessments

The primary endpoint corresponded to the description of the sonographic changes of the joints in paediatric patients with haemophilia based on the point-of-care ultrasound examination evaluated by HEAD-US method at baseline, 6, 12, 24 and 36 months after study entry. At baseline, patient demographics and medical, surgical and haemophilia history were collected. During each study visit (baseline, 6, 12, 24 and 36 months), investigators performed systematic joint assessments using HEAD-US protocol and HJHS v2.1 and collected information on number of bleeding episodes. Patients additionally completed PROs questionnaires for pain (Brief Pain Inventory [BPI]), functional ability (Haemophilia Activities List [HAL]/Paediatrics HAL [PedHAL]) and quality of life (Haemo-QoL) at each study visit.

As already mentioned, follow-up visits were prospectively scheduled at 6, 12, 24, and 36 months after baseline assessment. Due to the observational real-world design and varying recruitment times, not all patients completed all scheduled visits during the study period. For longitudinal analyses, comparisons were performed between baseline and each patient’s last available follow-up assessment.

The HEAD-US is an ultrasound-based scoring system used to detect and quantify joint damage in haemophilia patients. It assesses three key aspects of joint pathology: synovial hypertrophy (synovitis), cartilage damage, and subchondral bone changes. The elbows, knees and ankles were evaluated and scored based on synovitis (0–2), articular cartilage damage (0–4) and subchondral bone damage (0–2). Possible scores range from 0 to 8 per joint and therefore the total score ranges from 0 to 48, with higher scores indicating greater joint damage [9].The ultrasound examination was performed with a linear probe (emission range of 5–13 MHz) using a Samsung ultrasound system (Samsung Medison, Seoul, Republic of Korea) by an experienced investigator trained in the HEAD-US point-of-care ultrasound methodology, before implementation of the study protocol. Ultrasound assessment was performed according to the standardized HEAD-US protocol described by Martinoli et al. [9]. Elbows, knees, and ankles were systematically examined using predefined scanning planes and anatomical landmarks. Synovial hypertrophy was defined as abnormal hypoechoic intra-articular tissue; cartilage abnormalities included thinning and/or irregularity of the osteochondral surface, while subchondral bone changes included cortical irregularities and erosions. Power Doppler evaluation was performed when clinically indicated.

HJHS is a physical examination tool developed in 2006 by a consensus of experts for joint health assessment [10,11]. The HJHS is based on the examination of six index joints (elbows, knees and ankles) and gait assessment. There is a total score of 0−20 points per joint, in addition to the walking section which is scored from 0 to 4. Higher scores indicate poorer joint condition, ranging from 0 to 124 overall. The HJHS version 2.1 was used for the clinical assessment and was applied by experienced physiotherapists [20].

The Brief Pain Inventory (BPI) is a validated self-administered questionnaire widely used in clinical and research settings to evaluate pain severity and its impact on daily functioning [21]. It consists of 15 items and measures pain intensity on a 0–10 numerical rating scale and interference across seven domains, including general activity, walking, work, mood, enjoyment of life, relations with others, and sleep. Higher scores indicate greater severity and pain interference. Although this questionnaire was initially developed for the cancer pain population, it is now widely used for a variety of chronic pain presentations and has been translated and validated in many languages. The Greek version of the questionnaire was used in the present study [22].

The Paediatric Haemophilia Activities List (PedHAL) (version 0.12,2015) is a disease-specific, self- or proxy-administered questionnaire that includes 53 multiple-choice items across seven domains: sitting/kneeling/standing (10 items), functions of the legs (11 items), functions of the arms (6 items), use of transportation (3 items), self-care (9 items), household tasks (3 items), and leisure activities and sports (11 items) [23]. Each item is rated on a 6-point scale, from 1 (impossible) to 6 (never), describing difficulty due to haemophilia in the past month. Scores from individual items can be summed to give a total score or component scores for upper extremity, basic lower extremity, and complex lower extremity activities. Higher scores indicate greater functional status. The questionnaire also includes 4 multiple-choice items assessing the use of adaptive and assistive devices. There is a parent report version for children ages 4–8 and a child version (aged 8–18) with some minor linguistic differences.

The Haemophilia Quality of Life project, co-ordinated by the study centre at the University of Hamburg and co-operating with researchers from six European countries, started in 1998 and aimed at developing and testing a QoL assessment instrument for children and adolescents with haemophilia. The project ended in 2002 and has resulted in three sets of psychometrically tested questionnaire versions (Haemo-QoL) for three age groups of children (4–7, 8–12 and 13–16 years) as well as their parents, available in many languages, which are now ready for use in clinical and epidemiological trials [24,25,26]. The number of items varies by age group and dimensions typically include physical health, feelings, view, family, friends, perceived support, sports and school, coping, treatment, future, and relationships and others. The Haemophilia Quality of Life Questionnaire for Adults (Haem-A-QoL) was developed in 2005 and was initially validated in Italy [27].It consists of 46 items comprising 10 dimensions (physical health, feelings, view, sport and leisure time work and school, dealing, treatment, future, family planning, and relationships/partners). The psychometric properties of the Greek version of Haem-A-QoL instrument have been validated, and the instrument was found to be reliable and with good construct validity [28]. Scores by each dimension and total score in Haemo-QoL/Haem-A-QoL are transformed on scales ranging from 0 to 100, with 0 representing the best and 100 the worst HRQoL.

### 2.3. Statistical Analysis

Categorical variables were expressed as proportions, variables with a normal distribution were expressed as mean and standard deviation (SD) and variables with non-normal distribution were expressed as median and interquartile range (IQR). Mean HEAD-US scores for the six joints at baseline and last available follow-up visit were analyzed using repeated-measures ANOVA accounting for within-subject repeated joint measurements.The significance of main and interaction effects was evaluated, and post-hoc pair wise comparisons were conducted to assess differences between the two visits for each joint and between joints within each of the two visits. A second repeated-measure ANOVA was performed, replacing joint scores with scores for each of the three components of joint pathology assessed. Paired-samples t-tests were used to compare the number of bleeding episodes during the preceding 12 months, HJHS scores and PedHAL scores between first and last visit. All statistical analyses were conducted using standard statistical SPSS 28 software. A two-tailed *p*-value < 0.05 was considered statistically significant. Given the limited sample size and incomplete repeated observations across all scheduled visits, exploratory analyses comparing baseline with the last available follow-up assessment were preferred over mixed-effects longitudinal modeling.

## 3. Results

### 3.1. Study Population

Overall, the study enrolled 47 male children with haemophilia of all severities, with a mean age of 11.5 years (range 5–18). Most patients (n = 40) had haemophilia A, with 34 (85.0%) diagnosed as severe (FVIII < 1 IU/dL). Seven patients presented with haemophilia B, and five of these (71.4%) were classified as severe cases (FIX < 1 IU/dL). All patients underwent prophylactic therapy, the majority with extended half-life (EHL) FVIII or FIX concentrates. Additionally, a small subset of children with severe haemophilia A received emicizumab (n = 3). Among non-severe haemophilia patients (FVIII or FIX > 1–6 IU/dL), all exhibited a severe bleeding phenotype and were placed on prophylactic treatment following a joint bleed occurring before the age of five years. In total, 41 patients had HJHS/HEAD-US assessments at 6 months, 34 patients at 12 months, 18 at 24 months while six patients completed all four visits of the study. The baseline demographics and characteristics of the overall population are reported in Table 1. Time between visits refers to the interval between baseline and last available follow-up assessment.

The mean follow-up duration between baseline and last available assessment was 12.2 ± 5.0 months. Attrition increased over time, with only six patients completing all scheduled visits up to 36 months. Given that complete data across all visits were available for only a minority of the included patients, we evaluated the potential for attrition bias by comparing completers and non-completers regarding baseline characteristics and the parameters presented in Table 2 at the final visit. No statistically significant differences were identified between the two groups.

### 3.2. HEAD-US Ultrasound Evaluation

**Figure 1:**
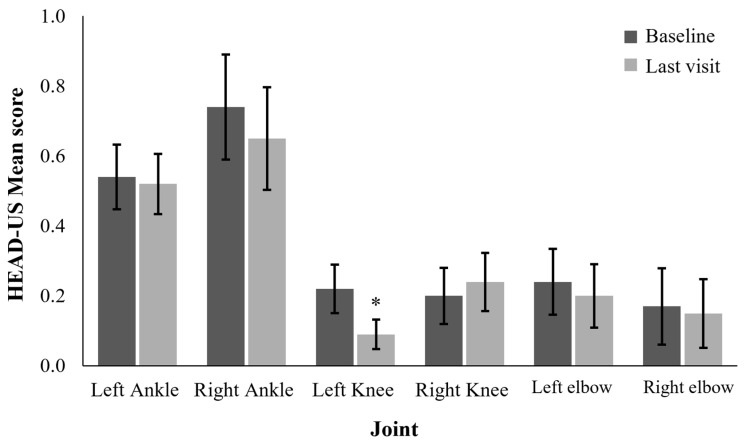
Mean values of the joint scores of the HEAD-US measurements at the first (baseline) and last visit (6, 12, 24 or 36 months). The error bars indicate the standard errors. The asterisk (*) denotes a *p* < 0.005. Maximum possible HEAD-US score per joint = 8.

In total, 252 joints of 47 patients underwent imaging evaluation using the HEAD-US procedure. Mean HEAD-US total scores were compared for all six joints between first visit (baseline) and last visit (6, 12, 24 or 36 months) (Figure 1). No significant effect of time on joint scores was observed (*p* = 0.229), while statistically significant differences were noted between the mean scores of the joints (*p* < 0.001). A statistically significant reduction in the mean HEAD-US total score was only seen for the left knee (baseline: 0.22; last visit: 0.09; *p* < 0.05). No statistically significant differences were seen for any other joints between baseline and last visit; however, baseline scores were already low, indicating minimal pre-existing joint damagein all assessed patients. At the joint level, the mean scores of the left and right ankles, both at the baseline and last visit were significantly greater than the mean scores of the rest of the joints (*p* < 0.05) (Figure 1).

**Figure 2:**
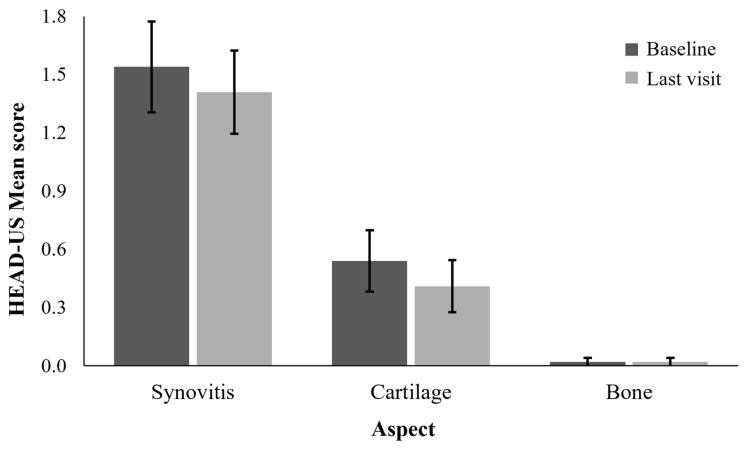
Mean values of the joint scores of the HEAD-US measurements for the three measured aspects (synovitis, cartilage, bone) at the first (baseline) and last visit (6, 12, 24 or 36 months). The error bars indicate the standard errors.

**Figure 3:**
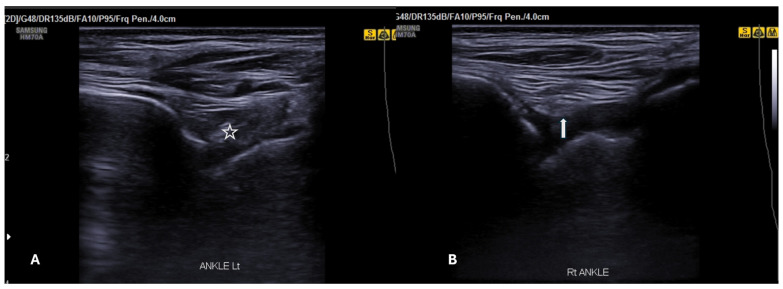
Example of point-of-care ultrasound with HEAD-US scoring system in a patient 17 years old with severe haemophilia A: Ventral longitudinal image plane of both the ankle joints, over the anterior tibiotalar recess. (**A**) Severe synovitis (star) fills the whole, of the recess volume (synovitis score 2). (**B**) Hypertrophic synovium (arrow) fills < 50%, of the recess volume (synovitis score 1).

Of the three components of joint pathology—synovial hypertrophy, cartilage damage, and subchondral bone changes, in terms of mean HEAD-US joint scores, synovitis contributed most to the total HEAD-US score, followed by cartilage damage (*p* < 0.001), whereas subchondral bone changes were minimal (*p* < 0.001) (Figure 2 and Figure 3). No statistically significant changes were reported between baseline and last visit mean HEAD-US joint scores in any of joint pathology components.

### 3.3. Clinical Joint Status

A total of 91.5% (n = 43/47) of patients and 258 joints were assessed and scored using the HJHS-2.1. The mean total HJHS-2.1, as assessed in the last visit (6, 12, 24 or 36 months), decreased compared to baseline, however the difference was not statistically significant (*p* = 0.611) (Table 2).

**Figure 4:**
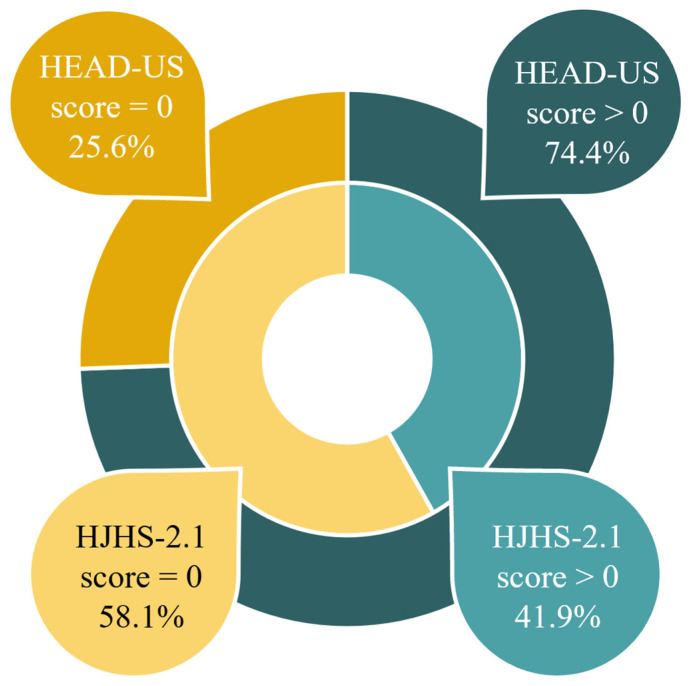
Distribution of joint scores at patient level in baseline between HJHS-2.1 (inner ring) and HEAD-US (outer ring). Percentages represent the relative share of zero and greater than zero scores within each assessment tool for the assessed patient population (N = 43).

When HJHS-2.1 and HEAD-US were compared at patient level at baseline, HEAD-US identified a greater number of children with non-zero scores, i.e., 74.4% (n = 32/43) in HEAD-US, vs.41.9% (n = 18/43) in HJHS, (Figure 4 and Table 3) and this finding was more prominent for the ankles.

### 3.4. Frequency of Bleeding and QoL

There was no statistically significant difference in the number of bleeding episodes in the last 12 months between baseline and last visit for all assessed patients (N = 47) (*p* = 0.366) (Table 2). Most bleeding episodes were mild joint bleeds managed conservatively, with no major haemorrhagic complications recorded during follow-up.

Overall, 42.6% (n = 20/47) of patients had at least two PedHAL evaluations, at baseline and at one of the study visits (6, 12, 24 or 36 months). The mean (±SD) overall score for paediatric (<18 years old) PedHAL was 93.9 (9.20) at baseline and 93.4 (12.80) at the last visit. Generally, high mean scores were observed for all PedHAL domains (indicating good functional ability).

The mean BPI score was 0 for all patients across in all visits.

Data related to the Haemo-QoL scores, collectively throughout the visits and categorized by age subgroup, are shown in Table 4. Mean scores were low overall across all ages, indicating a good overall QoL. Notably, adolescents (13–16 age subgroup) exhibited the lowest overall mean score (16.0), indicating low impairment due to haemophilia. The high SD depicted in the 4–7 age subgroup suggested heterogeneous experiences of QoL in early childhood, with some children likely experiencing low impairment, while others quite high. Patients in the 8–12 age subgroup had the highest overall mean score (24.1), though still not severely impaired.

Due to incomplete longitudinal availability of PRO data, QoL findings should be interpreted descriptively.

## 4. Discussion

The present study adds prospective real-world longitudinal paediatric data from a prophylaxis-treated cohort monitored using combined ultrasound, clinical examination, bleeding assessment, and patient-reported outcomes.

Despite major advances in prophylactic therapy, haemophilic arthropathy remains an important complication with long-term consequences. Early detection is therefore essential, and point-of-care tools help guide timely management to prevent or slow joint damage. Among these, HJHS and HEAD-US are the most widely used methods for assessing joint status.

The data presented in this study showed that paediatric haemophilia patients in Greece have an overall good clinical joint health in accordance with previous studies from other countries [29,30,31]. A multicenter study across Turkey with moderate or severe haemophilia patients ≥6 years of age only showed significant increases in HJHS and HEAD-US scores for the adult subpopulation at the end of a 12-month observation period, suggesting that prophylaxis in children delayed arthropathy progression [31]. These results concur with our observations that patients on baseline prophylaxis exhibited no significant changes in HJHS 2.1 or HEAD-US scores during follow-up. In a small Spanish cohort of 25 severe haemophilia A patients (4–19 years of age) on prophylaxis with no history of inhibitors, a well-preserved clinical joint status was recorded [29]. In another Spanish study of 82 patients aged ≥14 years with haemophilia B, those on prophylaxis had significantly better joint health (HEAD-US scores) than on-demand patients in all joints except the right ankle [30]. In a single-centre Italian study including 16 children with severe haemophilia on prophylaxis (aged 3 to 15 years), 12 months of sports participation did not result in significant changes in the already low baseline HJHS and HEAD-US scores [32]. A cross-sectional study of five European PedNet centres, including Greece, showed that most adolescents with moderate or severe haemophilia A maintain relatively favourable joint status under contemporary management based on HJHS and HEAD-US [16]. Finally, a recent HEAD-US study comparing adolescents with severe haemophilia on prophylaxis in Sweden and Greece reported generally good overall joint health in both countries, with earlier prophylaxis in Sweden associated with better joint outcomes [17].

The current analysis indicates that, at the joint level, ankles consistently exhibited higher mean HEAD-US scores relative to other joints. These findings reinforce the vulnerability of ankle joints as early targets of haemophilic arthropathy, in alignment with previous studies involving paediatric haemophilia patients in Spain (ages 4–19 on prophylaxis), Indonesia (ages 5–14, on-demand therapy), Turkey (ages 6–70 on prophylaxis), and Slovenia (ages 16–49 on prophylaxis) [15,29,31,33].

Synovitis contributed the most to the total mean HEAD-US scores throughout the study, in the present cohort, whereas cartilage damage was less prominent and subchondral bone changes were minimal. The low incidence of bone changes likely reflects early disease stage and effective prophylaxis. This pattern aligns with the recent European PedNet Registry study data showing synovial abnormalities as the most frequent finding in Swedish and Greek children with severe haemophilia [17]. In comparison, an earlier study with Nordic patients of all ages with moderate haemophilia A and B reported cartilage and bone as most affected according to HEAD-US [34]. The depicted difference could be attributed to the different populations assessed, considering the Nordic study included a predominantly adult population [median (IQR) age: 28 (13–52) years], whereas the present study focused on children and adolescents [mean (±SD) age, 11.5 (±3.7) years].

Baseline score comparison between HEAD-US and HJHS-2.1 at patient level showed superiority for HEAD-US in identifying children with non-zero scores. Across several studies, HEAD-US scores show positive correlations with HJHS however the strength varies by joint and cohort (children vs. adults, prophylaxis vs. episodic treatment) [12,15,18,34,35,36]. In populations such as children or well-managed patients receiving prophylaxis, including our cohort, disease severity may be mild and functional scores remain low. In these settings, ultrasound can detect early structural changes, which may not yet be reflected in functional impairment, thereby weakening the observed correlation [19,29]. Repeatedly, HEAD-US has been shown to be more sensitive for early anatomical changes that can be subclinical, whereas HJHS captures functional/physical impairments that sometimes occur without corresponding HEAD-US abnormalities [12,15,19,33]. Collectively, these findings support the complementary use of HEAD-US and HJHS for comprehensive joint monitoring in haemophilia across ages [29,35].

Early diagnosis of joint abnormalities can support timely adjustments in individual haemophilia treatment regimens [3], and recent studies show that systematic joint assessments, through functional examination combined with ultrasound could impact clinical decision-making in haemophilia care [37,38]. Integrating musculoskeletal ultrasound into routine assessment facilitates earlier detection of joint damage and enables more personalised prophylaxis optimization, ultimately supporting improved patient outcomes [37]. Musculoskeletal ultrasound offers several advantages in paediatrichaemophilia monitoring, including bedside availability, absence of ionizing radiation, rapid repeatability, and sensitivity for detecting subclinical synovial changes before irreversible structural damage develops [39,40].

A low baseline joint bleeding rate was recorded, and the number of bleeding episodes was not significantly affected throughout the study for all patients, similar to previous reports of children with haemophilia on prophylaxis [29,31].

Data from the health status and HRQoL PROs in the present cohort reflected low levels of impairment and a generally good overall QoL. Overall Mean Haemo-QoL scores were low across all age groups, with adolescents (13–16 years) reporting the lowest impairment (16.0), while children aged 8–12 years showed higher scores (24.1). This pattern may reflect adolescents’ greater coping skills, autonomy, and disease understanding, whereas younger children—whose activity levels are higher and coping strategies less developed—may have heightened symptom awareness and a stronger sense of limitations.

Limitations include the relatively small sample size, incomplete follow-up across all time points, and the absence of formal inter- and intra-observer reliability assessment for ultrasound measurements. However, haemophilia is a rare disease. The low baseline HEAD-US and HJHS scores likely introduced floor effects, limiting the ability to detect statistically significant longitudinal deterioration over time. Furthermore, attrition over time may have introduced selection bias, as patients completing longer follow-up may represent a subgroup with better adherence or milder disease phenotype. Additionally, parent-reported questionnaires in younger children may have been influenced by parental perception and expectations, potentially affecting HRQoL estimates. Our cohort included patients receiving different prophylactic regimens, including SHL, EHL products, and emicizumab, reflects contemporary real-world haemophilia practice, but may also have introduced treatment-related heterogeneity. Due to the limited sample size, subgroup analyses according to treatment type were not feasible. Additionally, handedness data were not systematically collected and therefore could not be reliably analyzed.

The strength of our database is the prospective non-interventional follow-up of children and adolescents treated prophylactically in our Centre, which is the largest paediatric haemophilia Centre in Greece.

## 5. Conclusions

In this cohort of children with haemophilia in Greece, joint health was well preserved under contemporary prophylaxis, with stable HJHS and HEAD-US scores and low bleeding rates throughout follow-up. Point-of-care Ultrasound proved more sensitive than clinical assessment for detecting early joint changes, underscoring its value as a complementary tool for longitudinal monitoring. Consistently, low Haemo-QoL, and high PedHAL scores in addition with zero BPI scores further reflected the favourable clinical status and overall good quality of life of participating patients. These findings support the integration of point-of-care ultrasound into routine paediatrichaemophilia care, enabling earlier detection of subclinical joint changes and facilitating individualized prophylaxis optimization. Further multicentre longitudinal studies with larger cohorts and standardized repeated imaging assessments are warranted.

## Figures and Tables

**Table 1:** Baseline demographics and characteristics.

	Overall Population (N = 47)		
Parameters	M [±SD]		
**Age at enrolment/baseline (years)**	11.5 [3.7]		
**Age at diagnosis (years)**	1.2 [1.3]		
**Severity of Haemophilia**	**n (%)**		
	**Overall** **(N = 47)**	**Type A** **(n = 40)**	**Type B** **(n = 7)**
Severe FVIII or FIX < 1%	39 (83.0)	34 (85.0)	5 (71.4)
Moderate FVIII or FIX = 1–5%	5 (10.6)	4 (10.0)	1 (14.3)
Mild FVIII or FIX 6–25%	3 (6.4)	2 (5.0)	1 (14.3)
**Number of study visits**	**n (%)**		
(Baseline)	47 (100)		
1	41 (87.2)		
2	34 (72.3)		
3	18 (38.3)		
4	6 (12.8)		
**Time between visits (months)**	12.2 [5.0]		
**≥1 target joint at enrolment/baseline**	n (%)		
Yes	4 (8.5)		
No	43 (91.5)		
**Treatment regimen at enrolment/baseline**	**n (%)**		
**Prophylaxis**	47 (100)		
Standard Half-Life Factors	16(34)		
Extended Half-Life Factors	28 (60)		
Emicizumab	3 (6)		
**On demand**	0 (0)		

M: Mean; SD: Standard deviation.

**Table 2:** HEAD-US score, HJHS-2.1 score, PedHAL score and frequency of bleeding.

	Overall Population (N = 47)		
Parameters	Baseline M [±SD]	Last Visit M [±SD]	*p* Value
Number of bleeding episodes during the last 12 months	0.65 [1.11]	0.50 [0.88]	0.366
HEAD-US score	2.1 [2.5]	1.9 [2.3]	0.229
HJHS-2.1 score ^†^	0.88 [1.33]	0.79 [1.36]	0.611
PedHAL score ^‡^	93.9 [9.20]	93.4 [12.80]	0.887

^†^ HJHS-2.1 scores were available for N = 43 patients, ^‡^ PedHAL scores were available for N = 20 patients. M: Mean; SD: Standard deviation; HJHS-2.1: Haemophilia Joint Health Score version 2.1; PedHAL: Paediatric Haemophilia Activities List.

**Table 3:** Proportion of children with non-zero scores in the HEAD-US measurements at baseline and last visit.

Overall Population (N = 43)				
Location	Measure	Baseline	Last Visit	*p* Value
Ankle	Synovitis	63.0%	60.9%	NS
Ankle	Cartilage and bone	19.6%	15.2%	NS
Knee	Synovitis	28.3%	26.1%	NS
Knee	Cartilage and bone	4.3%	4.3%	NS
Elbow	Synovitis	15.2%	13.0%	NS
Elbow	Cartilage and bone	10.9%	8.7%	NS

NS: Not significant.

**Table 4:** Haemo-QoL scores.

	Overall Population (N = 47)
Age 4–7	21.0 [20.30]
Age 8–12	24.1 [11.00]
Age 13–16	16.0 [9.10]

M: Mean; SD: Standard deviation; Haemo-QoL: Haemophilia Quality of Life Questionnaire.

## Data Availability

The raw data supporting the conclusions of this article will be made available by the authors on request.
